# Diet quality as assessed by the healthy eating index-2020 among different smoking status: an analysis of national health and nutrition examination survey (NHANES) data from 2005 to 2018

**DOI:** 10.1186/s12889-024-18630-7

**Published:** 2024-05-02

**Authors:** Ting Luo, Tung-Sung Tseng

**Affiliations:** 1 Moores Cancer Center, School of Medicine, University of California San Diego, 9500 Gilman Dr, 92093-0905, La Jolla, CA 92122 USA; 2 Behavioral and Community Health Sciences, School of Public Health, Louisiana State University Health Sciences Center-New Orleans, New Orleans, LA 70122 USA

**Keywords:** Healthy eating index (HEI-2020), Smoking status, Former smokers, Sodium intake, Weight control, NHANES

## Abstract

**Background:**

Combining smoking with poor eating habits significantly elevates the risk of chronic illnesses and early death. Understanding of how dietary quality shifts post-smoking cessation remains limited. The objective of this study is to examine dietary quality – using Healthy Eating Index (HEI – 2020) and its 13 components, among current, former, and never smokers, and particularly the impact of quitting and the duration of cessation on dietary habits.

**Methods:**

A cross-sectional analysis of 31,569 adults from the National Health and Nutrition Examination Survey (NHANES) 2005–2018 was conducted. Dietary quality was assessed using HEI-2020 scores, which were determined by NIH developed - simple HEI scoring algorithm per person. Smoking status was categorized into current, former, and never smokers, with further subdivisions for current (heavy/light smokers) and former smokers (duration post-cessation). Descriptive analysis and multiple regression models weighted to represent the US population were performed.

**Results:**

The current smoking rate was 19.4%, with a higher prevalence in males (22.5%) than females (17.5%). Current smokers reported statistically significantly lower HEI total score than both former and never smokers. Former smokers exhibited HEI scores similar to those of never smokers. The adjusted HEI total scores for current, former, and never smokers were 49.2, 54.0, and 53.3, respectively, with a statistically significant difference (*p* < 0.001). Moreover, light smokers had better total HEI score than heavy smokers (46.8 vs. 50.8, *p* < 0.001, respectively), but former and never smokers scored even higher. Quitting smoking immediately improved dietary quality, with former smokers reaching the dietary levels of never smokers within 5–10 years (53.8 vs. 53.3, *p* > 0.05, respectively). Compared to current smokers, former smokers tended to consume more beneficial foods (e.g., fruits, vegetables, greens and beans, whole grains, proteins, and fatty acids), while also consuming more sodium and less added sugar.

**Conclusions:**

Current smokers, particularly heavy smokers, exhibit poorer dietary habits than former and never smokers. The dietary quality of former smokers aligns with never smokers over time, highlighting the positive impact of smoking cessation on diet. This has implications for reducing chronic disease risks associated with poor diet and smoking.

**Supplementary Information:**

The online version contains supplementary material available at 10.1186/s12889-024-18630-7.

## Introduction

Smoking is a major risk factor for numerous diseases such as heart and respiratory diseases [1–3], and various cancers [4], with poor diet exacerbating these health issues [5]. Combined, smoking and unhealthy eating significantly raise the risk of chronic illness and early death [6, 7]. Tobacco use contributes to heart disease by reducing blood oxygen [1, 2], increasing blood pressure [1, 2], and damaging blood vessels [1, 2], risks that are increased by diets high in bad fats [8]. Additionally, the role of smoking in cancer development is worsened by diets lacking in fruits, vegetables, and fiber [4, 9]. Smoking and poor diet together also alters the composition of the gut microbiota, potentially leading to metabolic conditions like non-alcoholic fatty liver disease [10]. Quitting smoking not only enhances lung and heart health and reduces the risk of cancer, but it may also positively influence dietary habits [3]. Therefore, assessing dietary quality by smoking status, is crucial in understanding the impact of smoking cessation on nutritional habits, which may contribute to overall improved health outcomes.

The Healthy Eating Index (HEI), developed by the U.S. Department of Agriculture (USDA) and the National Cancer Institute (NCI), assesses dietary quality and alignment with the Dietary Guidelines for Americans, scoring from 0 to 100 [11]. Higher scores indicate better adherence to dietary guidelines [11, 12]. It includes components for various food groups and nutrients, with scores ranging from 0 to 100 [11]. The HEI comprises 13 components representing various food groups and nutrients, each rated on a scale up to either 5 or 10 points, where higher scores indicate better dietary compliance [11, 12].

Previous studies have reported that heavy smokers had significantly poorer dietary quality than those who never smoker, as assessed by dietary quality measurement tools such as Recommendation Compliance Index (RCI) and Diet Quality Index-International (DQI-I) [13]. A meta-analysis also revealed that smokers typically consume more energy, saturated fats, and cholesterol, but less antioxidant vitamins and fiber [14]. More existing literature has focused on specific food types, such as fruits and vegetables [15], fast food [16], food energy density [17], sugary beverages, and so forth, rather than evaluating broader indicators of overall diet quality, such as 13 HEI components. It has been consistently observed that current smokers consume fewer fruits and vegetables compared to non-smokers [15, 17].

However, only a few studies have examined dietary quality using HEI among current, former, and never smokers [18]. Reedy et al. reported the HEI-2015 scores differed between smokers and non-smokers, with the total score for current smokers (53.3) reporting much higher than nonsmokers (59.7). More specifically, former smokers scored higher in certain HEI components, such as total fruits, whole fruits, total vegetables, greens and beans, whole grains, sodium, while scoring lower in added sugar and saturated fats, compared to current smokers [18]. The higher sodium intake score (indicating lower intake) in current smokers remains unexpected, considering smoking is known to reduce taste sensitivity, often leading smokers to prefer stronger, saltier or sweeter flavors, which usually correlates with higher sodium consumption [19, 20].

Quitting smoking is undoubtedly a positive move for overall health, but it can introduce challenges related to diet [21, 22]. Nicotine suppresses appetite and increases metabolism, which can help smokers with weight control [23]. Yet, cessation often results in increased appetite and cravings, leading to potential weight gain [23]. Approximately half of women and one-third of men report hesitancy to quit or relapse due to weight concerns [24, 25]. Despite this, the long-term health benefits of quitting, like reduced disease risk, far outweigh the risk of weight gain [3]. Addressing weight concerns can involve a balanced diet, exercise, and craving management [26, 27]. However, the link between dietary quality and smoking status, particularly the impact of quitting and the duration of cessation on dietary habits, is not fully understood. Research is needed to investigate whether dietary quality improves over time after quitting and if it can eventually be comparable to that of never smokers. Therefore, the objective of this study is to assess dietary quality as assessed by the HEI-2020 across current, former, and never smokers, as well as to examine the effects of quitting and the duration of cessation on dietary habits.

## Methods

### Study design and data sources

National Health and Nutrition Examination Survey (NHANES) has been consistently gathering data since 1999, and it follows a two-year release cycle. This extensive data collection effort employs a complex, multistage sampling method that covers non-institutionalized civilians all over the US. The NHANES datasets offer an opportunity to evaluate dietary quality in relation to smoking status in the US. Utilizing data from NHANES spanning the years 2005–2006 to 2017–2018 allows for a detailed analysis of the interplay between dietary quality and smoking status, thanks to the comprehensive data range. In each survey cycle, approximately 10,000 individuals, representative of the US, are surveyed regarding their health, nutrition, and behaviors. To obtain large sample size and mitigate the year-to-year variability in healthy eating scores, we merged datasets from the seven NHANES cycles. The NHANES database is openly accessible to the public, containing de-identified information.

**Fig. 1:**
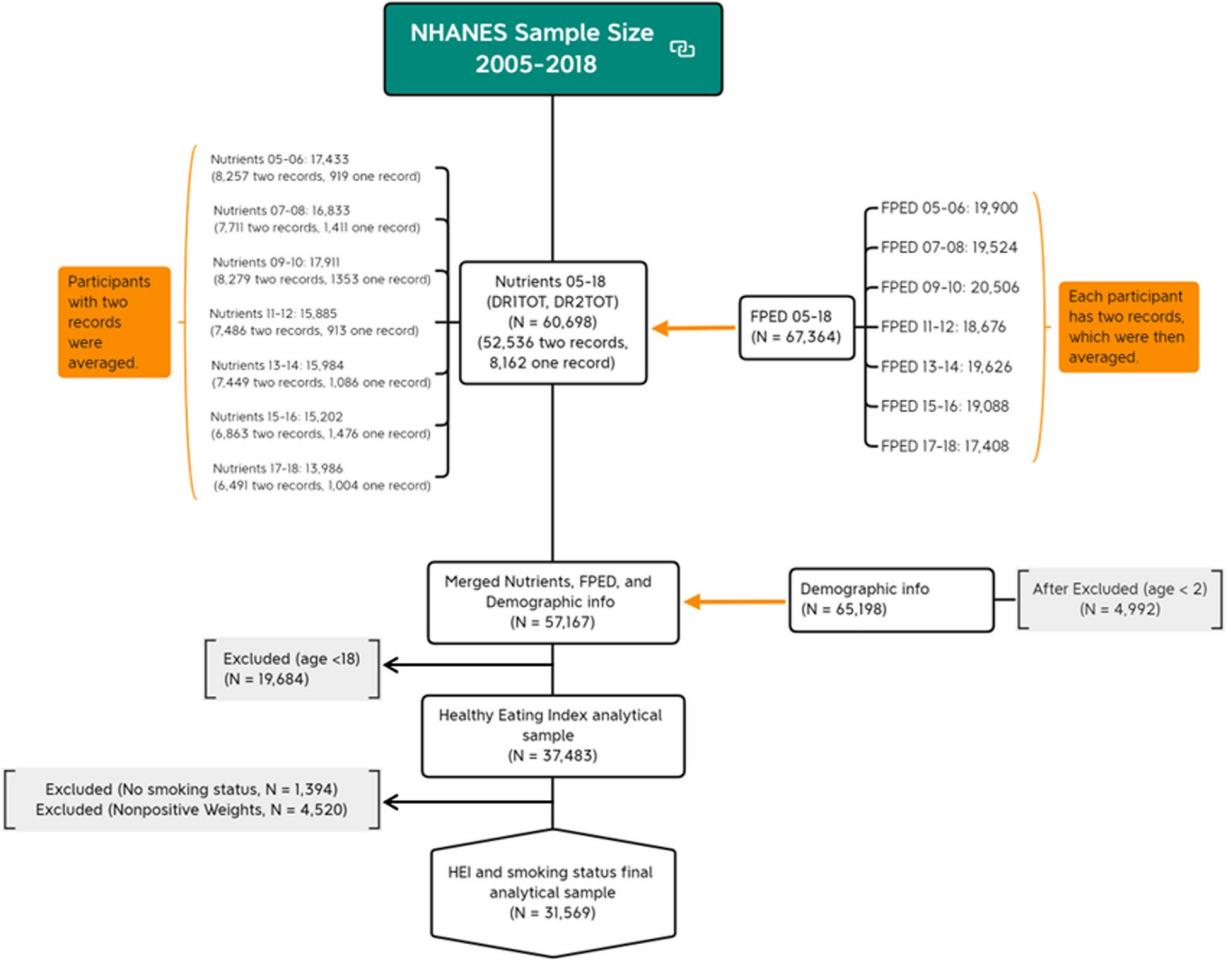
Flow chart of analytical sample screening

To convert NHANES dietary data into corresponding servings of major food groups and subgroups based on the HEI-2020 criteria, we accessed information from the US Department of Agriculture’s Food Pattern Equivalents Database (FPED) [28]. The dataset involving 67,364 participants. Each participant has two-day records, which were then averaged. NHANES nutrients datasets (DR1TOT and DR2TOT) provided 52,536 participants with two-day records, and 8,162 with one-day records. For those with two-day records, we calculated an average, while participants with only one day of records retained that single entry for our analysis. Combining information from FPED, nutrient datasets, and demographic data for individuals aged 2 and older (as dietary quality was assessed for those 2 and above) yielded a dataset encompassing 57,167 unique individuals. After excluding individuals under 18 years of age (*N* = 19,684, as smoking status was assessed for those aged 18 and above), those without smoking status information (*N* = 1,394), and individuals with non-positive weights (*N* = 4,520), our final analytical sample included 31,569 participants. This process is illustrated in Fig. 1, which outlines the dataset screening procedure.

### Measures

The primary outcome of this study was smoking status, categorized as current, former, or never smoker, as defined by the SMQ dataset. Using the benchmark of 100 lifetime cigarettes and current smoking: a “current smoker” has smoked at least 100 cigarettes and still smokes; a “former smoker” has smoked 100 or more cigarettes but has quit; and “never smokers” have either never smoked or smoked fewer than 100 cigarettes in total [29]. “Current smokers” were further divided into heavy (more than 10 cigarettes/day) and light (≤ 10/day). “Former smokers” were categorized by quit duration: 0–1 year, < 1–5 years, < 5–10 years, < 10–20 years, and 30 + years.

The NHANES employed a 24-hour recall interview to gather dietary intake data. The survey involved two 24-hour recalls: the first in-person and the second over the phone. This covered food names, consumption times, meal/snack types, and consumption locations. Visual aids and measurement tools were used to quantify reported foods and beverages. Trained interviewers conducted these assessments in a mobile examination center [28]. Mixed foods were separated into their ingredients by the National Cancer Institute (NCI) when creating FPED [28]. Diet quality was evaluated using HEI-2020, a USDA and NCI-developed measure aligning with Dietary Guidelines for Americans [11, 12]. It assigns scores ranging from 0 to 100, with higher scores indicating better diet quality. HEI-2020 comprises 13 components categorized as adequacy and moderation [11, 12]. Adequacy components assess the consumption of foods to encourage (higher scores reflect greater intake), including fruits, vegetables, whole grains, dairy, protein foods, and healthy fats. Moderation components evaluate the intake of foods to limit (higher scores indicate lower intake), such as saturated fats, refined grains, sodium, and added sugars [12].

Sociodemographic covariates included gender, age groups (18–40, 41–50, 51–60, or > 60 years), sex (male and female), race (Non-Hispanic White, Non-Hispanic Black, Hispanic, Non-Hispanic Asian, and other races), education (less than high school or more than high school), income level (IPR < 1.5, 1.5 ≤ IRP < 3.5 or IPR ≥ 3.5), health insurance coverage (Private insurance, Medicare or Medicaid, other health insurance, and no health insurance), marital status (Cohabiting, which includes married and living with a partner, vs. Other, which included widowed, divorced, separated, and never married). Body Mass Index (BMI) was categorized into three groups: Under/Normal weight (BMI < 24.9), Overweight (BMI 25.0 to 29.9), and Obesity (BMI 30+), with the underweight category merged into under/normal due to small proportion of participants. Physical activity levels were categorized as more than 150 min per week, 0–150 min per week, or none, based on adherence to CDC guidelines [30]. Binge drinking, depression, and Type II diabetes status were assessed as Yes or No. More specifically, binge drinking was defined as consuming five or more standard drinks for men and four or more for women within a two-hour timeframe. Depression was assessed using the commonly employed Patient Health Questionnaire-9 (PHQ-9), comprising nine questions, each rated on a scale of 0 to 3, resulting in a total score range of 0 to 27 [31, 32]. Scores between 0 and 9 were indicative of the absence of depression, while scores of 10 or higher were indicative of the presence of depression [31, 32]. Diabetes was defined as self-reported doctor-diagnosed diabetes or the use of diabetes medication (e.g. Glyburide) during the interview [33]. General health condition was classified as good (excellent, very good, and good) or poor (fair and poor) [34].

### Statistical analyses

To address the complexity of the sampling design, we incorporated commands tailored for survey data analysis. The analysis also accounted for the primary sampling unit, stratum for each observation, weighting, and data release number. Descriptive analyses described the characteristics of the study participants and the percentages of current, former, and never smokers. To calculate the HEI-2020 scores and its 13 components for each individual, we utilized the HEI-2015 scoring algorithm macro, originally developed by the NIH, given their identical components and scoring criteria [35]. Both unadjusted and adjusted HEI scores were presented. Multivariable regression analyses were adjusted for a range of variables, including adjusted for age, gender, race, education level, income ratio, marital status, physical activities, and depression status. The multivariable regressions passed the multicollinearity tests, with all VIF (Variance Inflation Factor) values below 5, tolerances close to 0, and the highest condition index below 25, indicating no significant multicollinearity in these models. All statistical analyses were conducted using SAS version 9.4, with a statistical significance set at *p* < 0.05.

## Results

Table 1 presents demographic, lifestyle, and health factors among 31,569 participants, categorized by their smoking status: current (6,138, 19.4%), former (7,713, 24.4%), and never (17,718, 56.2%). The current smoking rate was 19.4%, with 22.5% among males and 17.5% among females. Significant differences were observed across all categories. Age distribution varied, with most current smokers (40.5%) in the 51–60 age group, while most former smokers (40.0%) in the > 60 age group. The mean age for former smokers was 54.1 years, which was 11.4 years higher than current smokers and 8.8 years older than never smokers. Males were more prevalent among current (53.5%) and former smokers (56.7%), while never smokers had more females (58.1%). Non-Hispanic Whites dominated all categories but were most prominent among former smokers (74.2%). Education seemed to inversely correlate with current smoking, with a higher percentage of those with only a high school education or below being current smokers (56.4% vs. 43.6%). Individuals with lower income to poverty ratio (IPR < 1.5) had a relatively high current smoking rate of 40.3%. Additionally, characteristics like private insurance, cohabitation, overweight and obesity, lack of physical activity, non-binge drinking, no depression, absence of diabetes, and good health condition were predominant in never and former smokers.

**Table 1:** Demographic, lifestyle, and health characteristics by smoking status among US population aged ≥ 18 years (*N* = 31,569)

Variables	Overall (N, %)	Current Smoker (*N* = 6,138, 19.4%) % (95% CI)	Former Smoker (*N* = 7713, 24.4%) % (95% CI)	Never Smoker (*N* = 17,718, 56.2%) % (95% CI)	*P*-value
Age (Mean, SD)^a^	47.0 (0.20)	42.7 (0.26)	54.1 (0.41)	45.3 (0.26)	< 0.001
Age					< 0.001
18–40	6217 (21.7)	25.6 (23.9–27.3)	10.2 (8.9–11.6)	25.2 (23.8–26.7)	
41–50	5151 (17.9)	20.8 (19.3–22.3)	13.1 (12.0-14.3)	18.9 (18.0-19.9)	
51–60	10,326 (36.3)	40.5 (38.7–42.4)	36.7 (34.9–38.4)	34.7 (33.4–36.0)	
> 60	9875 (24.2)	13.1 (11.9–14.3)	40.0 (38.0-41.9)	21.1 (19.9–22.3)	
Gender					< 0.001
Male	15,014 (47.7)	53.5 (51.7–55.3)	56.7 (55.1–58.3)	41.9 (40.7–43.0)	
Female	16,555 (52.3)	46.5 (44.7–48.3)	43.3 (41.7–44.9)	58.1 (57.0-59.3)	
Race					< 0.001
NH-White	6878 (64.6)	65.1 (60.7–69.6)	74.2 (70.8–77.5)	60.5 (56.8–64.1)	
NH-Black	4120 (11.4)	15.2 (12.4–18.0)	7.2 (5.8–8.7)	12.0 (9.9–14.2)	
Hispanic	4144 (15.1)	11.6 (9.1–14.0)	12.1 (9.9–14.3)	17.4 (14.6–20.2)	
NH-Asian	1975 (5.5)	2.4 (1.7–3.1)	3.2 (2.4-4.0)	7.4 (6.1–8.6)	
Other race	670 (3.4)	5.7 (4.4–6.9)	3.3 (2.6-4.0)	2.7 (2.2–3.3)	
Educational level					< 0.001
High school or below	14,298 (38.3)	56.4 (54.1–58.6)	38.0 (35.8–40.3)	32.1 (30.4–33.8)	
More than high school	16,575 (61.7)	43.6 (41.4–45.9)	62.0 (59.7–64.2)	67.9 (66.2–69.6)	
Income to Poverty Ratio					< 0.001
IPR < 1.5	10,245 (25.1)	40.3 (37.6–43.0)	20.3 (18.6–22.0)	22.0 (20.5–23.5)	
1.5 = < IPR = < 3.5	9539 (31.7)	33.0 (30.5–35.5)	33.0 (31.1–35.0)	30.7 (29.1–32.3)	
IPR > 3.5	8987 (43.2)	26.7 (24.1–29.3)	46.7 (44.1–49.2)	47.3 (45.0-49.6)	
Insurance type					< 0.001
Private	16,611 (61.8)	43.9 (41.5–46.3)	65.0 (62.8–67.3)	66.5 (64.9–68.2)	
Medicare/Medicaid	6282 (14.5)	18.7 (17.3–20.2)	17.1 (15.7–18.5)	11.9 (10.9–12.8)	
Other insurance	2117 (6.4)	7.9 (6.7-9.0)	5.5 (4.7–6.3)	6.2 (5.6–6.9)	
No insurance	6432 (17.4)	29.5 (27.3–31.6)	12.3 (10.8–13.9)	15.4 (14.2–16.6)	
Marital status					< 0.001
Cohabiting	18,662 (63.3)	53.6 (51.2–56.0)	68.6 (66.9–70.4)	64.3 (62.8–65.7)	
Other	12,224 (36.7)	46.4 (44.0-48.8)	31.4 (29.6–33.1)	35.7 (34.3–37.2)	
BMI					< 0.001
Under/Normal weight	8799 (30.0)	36.2 (34.3–38.0)	23.5 (21.8–25.1)	30.8 (29.4–32.2)	
Overweight	10,189 (32.9)	30.4 (28.6–32.3)	36.3 (34.5–38.1)	32.2 (31.0-33.4)	
Obesity	11,843 (37.1)	33.4 (31.4–35.4)	40.2 (38.3–42.2)	37.0 (35.5–38.5)	
Moderate-intensity work					< 0.001
> 150 min per week	7862 (32.7)	39.0 (36.9–41.0)	32.8 (30.7–34.9)	30.5 (29.0–32.0)	
0–150 min per week	2286 (9.4)	7.5 (6.5–8.5)	10.7 (9.4–12.0)	9.4 (8.5–10.2)	
None	17,286 (58.0)	53.5 (51.6–55.4)	56.5 (54.2–58.8)	60.1 (58.5–61.6)	
Binge drinking					< 0.001
Yes	2208 (8.5)	19.9 (17.9–21.9)	7.1 (6.2–8.1)	5.3 (4.6–5.9)	
No	24,244 (91.5)	80.1 (78.1–82.1)	92.9 (91.9–93.8)	94.7 (94.1–95.4)	
Depression					< 0.001
Yes	2615 (8.1)	17.0 (15.3–18.7)	6.7 (5.7–7.8)	5.6 (5.1–6.2)	
No	27,194 (91.9)	83.0 (81.3–84.7)	93.3 (92.2–94.3)	94.4 (93.8–94.9)	
Diabetes					< 0.001
Yes	4047 (9.4)	7.4 (6.5–8.4)	13.9 (12.6–15.2)	8.0 (7.4–8.6)	
No	27,503 (90.7)	92.6 (91.6–93.5)	86.1 (84.8–87.4)	92.0 (91.4–92.6)	
General health condition					< 0.001
Good	23,002 (82.8)	73.1 (71.6–74.6)	82.2 (80.8–83.7)	86.3 (85.4–87.3)	
Poor	7007 (17.2)	26.9 (25.4–28.4)	17.8 (16.3–19.2)	13.7 (12.7–14.6)	

“%” refers to weighted %

Table 2 provides the unadjusted and adjusted HEI-2020 total scores and its 13 component scores categorized by smoking status. In the unadjusted data, the total HEI score for former smokers (55.30) closely mirrors that of never smokers (54.93), yet statistically significantly surpassed the score for current smokers (47.75). Upon adjusting for potential covariates, the differences between former smokers and never smokers narrowed compared to never smokers, but remained huge (53.96 vs. 53.33 vs. 49.18, respectively). Current smokers consistently showed lower total HEI score than former and never smokers, indicating poorer overall diet quality among current smokers. Former and never smokers had close scores, suggesting similar diet quality regardless of past smoking status as long as they are quitting smoking. Specifically, in terms of 13 components, both unadjusted and adjusted models consistently indicated that current smokers tended to consume fewer beneficial foods, such as total vegetables, greens and beans, total fruits, whole fruits, whole grains, total diary, total protein, sea and plants and proteins, and fatty acid. Conversely, they exhibited increased consumption of foods recommended in moderation, notably added sugars. Interestingly, higher scores (representing lower sodium intake) were also found in both models.

**Table 2:** HEI-2020 total and component scores by smoking status among US population aged ≥ 18 years (*N* = 31,569)

HEI and components	Current smoker	Former smoker	Never smoker
**Unadjusted**			
HEI − 2015 total score (0 -100)	47.75 (47.15–47.15)	55.30 (54.68–54.68)	54.93 (54.45–54.45)
Total Vegetables (0–5)	2.86 (2.80–2.80)	3.42 (3.37–3.37)	3.40 (3.36–3.36)
Greens and Beans (0–5)	1.49 (1.40–1.40)	2.09 (1.98–1.98)	2.13 (2.07–2.07)
Total Fruits (0–5)	1.54 (1.46–1.46)	2.49 (2.41–2.41)	2.62 (2.56–2.56)
Whole Fruits (0–5)	1.53 (1.44–1.44)	2.77 (2.68–2.68)	2.76 (2.69–2.69)
Whole Grains (0–10)	1.89 (1.78–1.78)	3.07 (2.94–2.94)	2.96 (2.87–2.87)
Dairy (0–10)	4.99 (4.86–4.86)	5.36 (5.25–5.25)	5.51 (5.41–5.41)
Total Protein Foods (0–5)	4.37 (4.32–4.32)	4.55 (4.52–4.52)	4.52 (4.49–4.49)
Seafood and Plant Proteins (0–5)	2.33 (2.25–2.25)	3.05 (2.97–2.97)	3.01 (2.95–2.95)
Fatty Acids (0–10)	4.36 (4.21–4.21)	5.01 (4.88–4.88)	5.02 (4.94–4.94)
**Sodium (0–10) ¶**	4.61 (4.47–4.47)	3.92 (3.81–3.81)	3.97 (3.89–3.89)
**Refined Grains (0–10) ¶**	6.42 (6.29–6.29)	6.56 (6.44–6.44)	6.01 (5.91–5.91)
**Saturated Fats (0–10) ¶**	5.76 (5.61–5.61)	5.72 (5.59–5.59)	6.00 (5.91–5.91)
**Added Sugars (0–10) ¶**	5.60 (5.44–5.44)	7.29 (7.18–7.18)	7.03 (6.93–6.93)
**Adjusted**			
HEI − 2015 total score (0 -100)	49.18 (48.18–50.18)	53.96 (52.79–55.12)	53.33 (52.51–54.16)
Total Vegetables (0–5)	2.80 (2.71–2.90)	3.16 (3.06–3.25)	3.19 (3.10–3.28)
Greens and Beans (0–5)	1.70 (1.54–1.87)	2.13 (1.97–2.29)	2.08 (1.95–2.22)
Total Fruits (0–5)	1.66 (1.49–1.82)	2.32 (2.18–2.47)	2.45 (2.31–2.58)
Whole Fruits (0–5)	1.69 (1.49–1.89)	2.55 (2.37–2.72)	2.55 (2.40–2.69)
Whole Grains (0–10)	2.08 (1.87–2.30)	2.91 (2.66–3.16)	2.78 (2.61–2.95)
Dairy (0–10)	4.50 (4.23–4.77)	4.80 (4.55–5.06)	4.94 (4.73–5.15)
Total Protein Foods (0–5)	4.38 (4.30–4.45)	4.50 (4.44–4.56)	4.48 (4.41–4.54)
Seafood and Plant Proteins (0–5)	2.55 (2.38–2.72)	2.99 (2.84–3.13)	2.96 (2.83–3.10)
Fatty Acids (0–10)	4.78 (4.50–5.05)	5.37 (5.08–5.67)	5.21 (4.98–5.44)
**Sodium (0–10) ¶**	4.61 (4.31–4.91)	3.85 (3.60–4.11)	3.95 (3.76–4.15)
**Refined Grains (0–10) ¶**	6.30 (6.06–6.55)	6.13 (5.88–6.37)	5.74 (5.56–5.93)
**Saturated Fats (0–10) ¶**	6.17 (5.89–6.45)	6.06 (5.78–6.35)	6.14 (5.91–6.38)
**Added Sugars (0–10) ¶**	5.96 (5.69–6.23)	7.18 (6.95–7.41)	6.85 (6.62–7.09)

Higher scores align more closely with dietary guidelines and reflect better dietary choices. For sodium, refined grains, saturated fats, and added sugar marked with the symbol “¶”, higher scores indicate lower consumption; whereas for the remaining 9 components, higher scores indicate greater consumption. Adjusted model: adjusted for age, gender, race, education level, income ratio, marital status, physical activity, and depression status

In order to further investigate the difference between smoking status, we conducted regression analysis. Similar trends were observed in both the crude and adjusted models. The adjusted models, controlling multiple variables, strengthened the evidence for the robustness of the observed relationship. Table 3 presents adjusted estimates for the comparison of the any pair of smoking statuses. In the upper section, we reported the HEI and its 13 components. Former smokers exhibited similar scores across the total HEI and its 11 components, except for refined grains and added sugar, which had significantly higher scores (indicating lower intake). Compared to current smokers, former and never smokers had 4.78 and 4.16 higher points in HEI total score, respectively, and reported higher consumption of nine beneficial food items, including total vegetables, greens and beans, whole fruits, whole grains, total protein, seafood and plant proteins, and healthy fatty acids. However, they also reported higher consumption of added sugar (to be limited) and lower consumption of sodium (to be limited).

**Table 3:** Comparing HEI − 2020 and its 13 components and nutrients intake across different smoking status in adjusted models

	Former vs. Current	Never vs. Current	Former vs. Never
**HEI and Components**			
HEI − 2015 total score (0 -100)	4.780***	4.160***	0.622
Total Fruits (0–5)	0.356***	0.388***	-0.032
Whole Fruits (0–5)	0.427***	0.382***	0.045
Total Vegetables (0–5)	0.670***	0.790***	-0.121
Greens and Beans (0–5)	0.860***	0.861***	-0.001
Whole Grains (0–10)	0.827***	0.697***	0.130
Dairy (0–10)	0.305**	0.445***	-0.140
Total Protein Foods (0–5)	0.119**	0.097*	0.022
Seafood and Plant Proteins (0–5)	0.436***	0.415***	0.021
Fatty Acids (0–10)	0.594***	0.431***	0.163
Sodium (0–10) ¶	-0.753***	-0.654***	-0.099
Refined Grains (0–10) ¶	-0.176	-0.560***	0.384***
Saturated Fats (0–10) ¶	-0.107	-0.026	-0.081
Added Sugars (0–10) ¶	1.220***	0.891***	0.331**
**Nutrients Intake and food variety**			
Total Energy (kcal)	-71.63*	-63.05*	-8.590
Carbohydrates (gm)	-10.52*	0.232	-10.80**
Protein (gm)	3.680***	4.100***	-0.418
Total Fat (gm)	1.490	0.379	1.107
Total saturated fatty acids (gm)	-0.493	-0.527	0.034
Total monounsaturated fatty acid (gm)	0.854	0.154	0.700
Total polyunsaturated fatty acid (gm)	1.210**	0.733*	0.481
Fiber (gm)	2.630***	2.860***	-0.228
Number of Foods	1.650***	1.340***	0.313

For sodium, refined grains, saturated fats, and added sugar marked with the symbol “¶”, higher scores indicate lower consumption; whereas for the remaining 9 components, higher scores indicate greater consumption. Adjusted model: adjusted for age, gender, race, education level, income ratio, marital status, physical activity, and depression status. Significant level in this table: *p < 0.05, **p < 0.01, ***p < 0.001

In the lower section, the nutrients intake and food variety were reported. Compared to current smokers, both former and never smokers reported lower total energy intake (-71.63 kcal and − 63.05 kcal, respectively) and higher intake of protein (3.68gm and 4.10gm, respectively), total polyunsaturated fatty acids (1.21gm and 0.73gm, respectively), and fiber (2.63gm and 2.86gm, respectively), and a wider variety of foods (1.65 and 1.34, respectively). Notably, former smokers also reported lower carbohydrate intake (-10.52gm) compared to current smokers, a difference not observed in the comparison between never smokers and current smokers. Former smokers displayed a dietary pattern closely resembling that of never smokers in terms of macronutrients, fiber intake, and food variety. The only notable distinction was a significant increase in carbohydrate intake among former smokers, amounting to an additional 10.8 g.

**Fig. 2:**
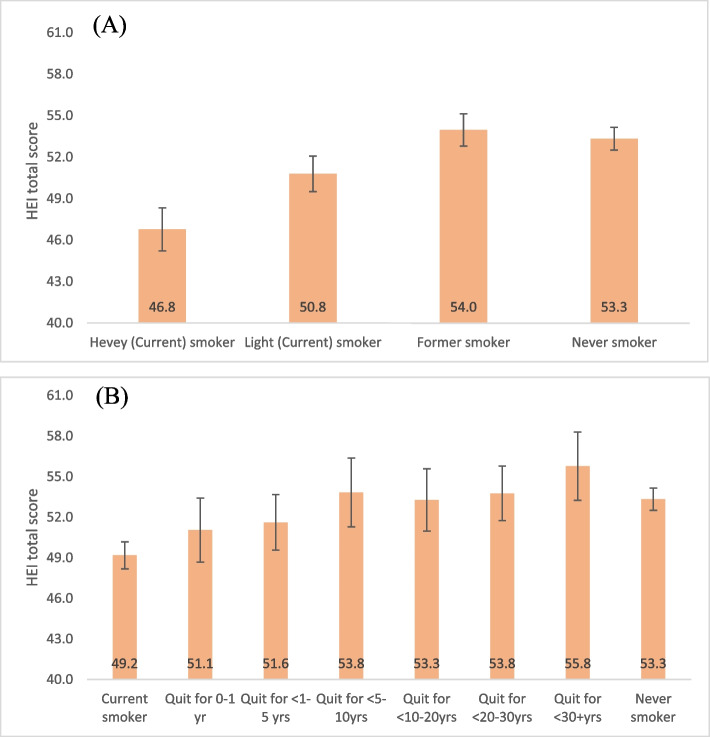
Subgroup comparison in HEI-2020 total score by smoking status and quitting duration. HEI score adjusted for age, gender, race, education level, income ratio, marital status, physical activity, and depression status. Heavy current smokers: >10 cigarettes/day; Light current smokers: ≤10 cigarettes/day. Quit durations: 0–1 year (0-365 days), < 1–5 yrs (< 365–1825 days), < 5–10 yrs (< 1825–3650 days), < 10–20 yrs (< 3650–7300 days), < 20–30 yrs (< 7300–10,950 days), > 30 yrs (> 10,950 days)

By closely examining the variances in the HEI-2020 total score, we further categorized current smokers into light and heavy smokers, and former smokers into groups based on their quitting duration: 0–1 year, < 1–5 years, < 5–10 years, < 10–20 years, and 30 + years. Figure 2 (A) shows that light smokers reported higher HEI total scores than heavy smokers (50.8 vs. 46.8, *p* < 0.001, respectively), and former smoker and never smoker displayed even higher HEI total score than light smokers (former 54.0 vs. light 50.8, *p* < 0.001; never 53.3 vs. light 50.8, *p* < 0.001). Figure 2 (B) provides a detailed analysis based on the time since quitting smoking, including current smokers, those who quit for durations ranging from 0 to 1 year to over 30 years, and never smokers. A noticeable trend suggested that scores improved with the length of time since quitting (*P* < 0.05). Those who quit smoking in the 5–10 year range had significantly higher HEI total scores than current smokers (53.8 vs. 49.2, *p* < 0.001). Even within the first year of quitting, individuals showed better HEI scores than current smokers, though the difference was not statistically significant (51.1 vs. 49.2, *p* > 0.05). Former smokers who quit between 5 and 10 years also reported similar HEI total scores to never smokers (53.8 vs. 53.3, *p* > 0.05, respectively).

## Discussion

We utilized the nationally representative NHANES datasets from 2005 to 2018 to confirm our hypothesis regarding the association between dietary quality assessed by HEI scores and its 13 components among smoking status, categorizing individuals as current, former, and never smokers. Additionally, this study delved into the impact of smoking cessation duration on dietary habits, providing insights into the link between dietary quality and smoking status.

Our analysis found that 19.4% of adults aged ≥ 18 were current smokers, with a higher prevalence in men (22.5%) compared to women (17.5%). Between 2005 and 2019, cigarette smoking rates ranged from 20.9 to 14.0%, with men ranging from 23.9 to 15.3% and women from 18.1 to 12.7%, and our results fall in this range [36–39]. Current smokers were more prevalent among men, those with lower education, lower income, non-cohabiting individuals, and those without private insurance, consistent with previous CDC findings [36–39]. Our data also showed that smokers scored 4.78-point lower on the HEI total score than former smokers in adjusted model, affirming previous research [18]. The consistency with previous studies indicates the reliability of our analysis.

Our study also showed that former and never smokers typically had a better diet quality than current smokers, in line with previous research [13–15, 17, 18, 40, 41]. On the one hand, current smokers in our study reported consuming a higher amount of energy and carbohydrates (a primary source of energy) but less protein than former smokers. This pattern suggests a preference for energy-dense foods, which are often high in carbohydrates and fats but lower in protein. While no significant difference in saturated fat intake was noted between former and current smokers, the former group reported higher consumption of total polyunsaturated fatty acids (a healthier fat type).

On the other hand, current smokers were found to consume fewer fruits, vegetables, greens and beans, whole grains, and varied proteins, while having a higher intake of added sugars. Previous studies have reported that smokers tended to consume less vitamin C-rich fruits and vegetables, although they require more vitamin C to counteract the free radicals from smoking [18, 20, 40, 42–44]. On average, one cigarettes destroys about 20 mg of vitamin C [42], which has also been associated with lower serum vitamin C levels in smokers [41]. Moreover, our supplementary analysis of data spanning from the 2007–2008 to the 2017–2018 cycles revealed that current smokers exhibited lower vitamin C supplement intake (228.6 mg) compared to former smokers (244.7 mg) and never smokers (224.2 mg) (as displayed in supplementary Table 1). Furthermore, it is worth noting that vitamin E’s antioxidant function is compromised in the absence of vitamin C [43]. Considering the increased risk of chronic diseases resulting from the combination of smoking and poor dietary quality, with approximately 30 million current smokers in the US [36–39], it is important to continue raising awareness about the intake of fruits and vegetables for smokers. Further research is necessary to better understand and address the dietary challenges faced by smokers.

Moreover, our study revealed an unexpected finding regarding sodium intake, challenging previous research suggesting that smoking might influence taste perception and lead to a preference for saltier foods, implying higher sodium intake among current smokers [19, 20]. Instead, our findings indicated that current smokers reported lower sodium intake compared to former or never smokers. Actually, our supplementary analysis showed that the percentage of current smokers following a low-salt diet was not higher than former and never smokers (1.6% vs. 2.4% vs. 1.5%, respectively). We also found that a higher percentage of current smokers (40.6%) often used salt during cooking than former (34.5%) and never smokers (37.1%). Similarly, more current and former smokers used table salt recently (36.0% and 37.0%) than never smokers (29.2%). These additional findings support the idea that current smokers do not necessarily prefer less salt diets, in line with previous research [19, 20]. The reduced sodium intake observed in current smokers might result from their lower overall food consumption. The appetite-suppressing by nicotine effect may lead smokers to consume fewer meals than others [41]. Our study echoed this, as current smokers consumed fewer food items. While they had a statistically significantly higher energy intake in the unadjusted model, their diets were characterized by lower protein, higher carbohydrates (with higher sugar), and less fiber, suggesting a preference for high-energy-density or empty-calorie foods, which may contribute to their lower sodium intake.

Similarly, our study found no significant difference in saturated fat intake across current, former, and never smokers, deviating from prior research linking smoking to higher saturated fat consumption [15]. This variation might stem from the overall lower food intake reported by current smokers, aligning with previous studies suggesting that smokers generally consume less food [15, 41]. Consequently, this reduced consumption could naturally lead to lower saturated fat intake. Supplementary Table 2 in our additional analysis corroborates this: current smokers tended to have a less varied diet compared to former and never smokers (13.8 vs. 15.4 vs. 15.1, *p* < 0.001). Prior meta-analysis indicated that dietary variety significantly influenced food intake [45]. This can be particularly relevant when considering that smoking may impair taste bud function, potentially diminishing the appeal of food and resulting in decreased consumption.

Another significant finding was that light current smokers reported higher HEI total score than heavy smokers, and quitting smoking improves dietary quality than smoking, evident within the first year. This improvement could be due to restored taste perception, leading to healthier food choices for former smokers [46]. Moreover, former smokers who have abstained for 5–10 years had dietary scores similar to never smokers suggests that former smokers’ dietary behaviors tend to normalize over time. Quitting smoking not only improves lung health and reduces cancer risk as previous studies reported but also leads to better dietary choices [1–4].

However, current smokers are often concerned about weight gain after quitting, which can decrease their motivation to quit. It is worth noting that while former smokers, reported a higher obesity rate than both never smokers and current smokers (40.2% vs. 37.0% vs. 33.4%, respectively), a closer examination by quitting duration revealed a more nuanced picture. Specifically, among former smokers categorized by quitting duration (0–1 year, < 1–5 years, < 5–10 years, < 10–20 years, and 30 + years), the obesity rates were 38.7%, 42.3%, 37.1%, 43.3%, 42.7%, and 37.5%, respectively (see Supplementary Table 1). These findings indicate that the obesity rate does not significantly rise in the first year after quitting but increases between 1 and 5 years, and those who quit for 30 years have obesity rates similar to never smokers. It is also worth noting that the energy intake remained relatively consistent across different quitting durations; however, a decline in moderate-to-intense physical activity over time was noted, which could contribute to weight gain (see Supplementary Table 1). Thus, increasing physical activity and controlling energy intake may help for weight management. Previous research supports that regular weight monitoring and adjustments in diet and exercise are beneficial [26, 27]. Therefore, while quitting smoking might lead to initial weight gain, it can be managed with lifestyle adjustments.

### Limitations and strengths

The present study exhibits several limitations. Firstly, its cross-sectional design prevents the establishment of causal relationships between HEI and smoking status. Secondly, the reliance on self-reported dietary intake data introduces the potential for recall bias and measurement errors, which could impact the accuracy of the results. Similarly, self-reported smoking behavior may also be susceptible to recall bias. Furthermore, the HEI calculation method adapted in this study is simple HEI scoring algorithm – per person. While this approach may not yield the most precise estimates of means with least biased as it does not account for the relationship between HEI components, it aligns with the objective of this study of gaining a broad understanding of the dietary habits within a specific population [35, 47]. This simplified HEI scoring algorithm per person effectively serves the purposes of our research.

Despite these limitations, the study has some strengths that enhance our understanding of dietary habits among individuals who are current, former, and never smokers. The utilization of a nationally representative dataset like NHANES bolsters the study with a robust sample size, thereby enhancing the external validity of the findings. Moreover, NHANES covers a wide spectrum of areas, enabling us to account for various factors that influence these associations, including sociodemographic factors, behavioral and lifestyle choices, psychological aspects, and chronic health conditions, thereby reinforcing the robustness of our analysis. Furthermore, the focus of this study on different current and former smoker subgroups holds potential value for public health practitioners and policymakers in formulating effective strategies for tobacco cessation initiatives.

## Conclusions and implications

In conclusion, current smokers reported statistically significantly lower HEI total score (49.0) compared to former (54.0) and never smokers (49.0), with former smokers showing similar HEI total score to never smokers. Current smokers typically consume energy-dense foods, notably less of beneficial foods (e.g., fruits, vegetables, greens and beans, whole grains, dairy, proteins, and fatty acids), while having higher intake of added sugars and lower sodium intake than former and never smokers. A dose-response relationship was observed with dietary quality: light smokers had better dietary quality than heavy smokers, but former and never smokers had even better dietary quality. Quitting smoking immediately improved dietary quality, with former smokers reaching the dietary levels of never smokers within 5–10 years.

This evidence underscores the importance of integrating smoking cessation efforts with dietary guidance, highlighting the dual benefits for enhancing overall health outcomes. The dose-response relationship between smoking intensity and dietary quality emphasizes the need for targeted interventions for heavy smokers, who are at higher risk of poor nutrition and related health issues. It advocates for comprehensive health promotion strategies that address multiple risk factors simultaneously, offering a more holistic approach to disease prevention and health improvement.

### Supplementary Information

**Supplementary Material 1.**

## Data Availability

Data were available from the NHANES website: https://wwwn.cdc.gov/nchs/nhanes/Default.aspx. The analytic code used to conduct the analyses presented in this study is not available in a public archive but may be obtained by emailing the corresponding author.
