# Supplementary material for: Diet quality as assessed by the healthy eating index-2020 among different smoking status: an analysis of national health and nutrition examination survey (NHANES) data from 2005 to 2018
Source: BMC Public Health. 2024 May 2;24:1212. doi: 10.1186/s12889-024-18630-7 (PMC11064397; doi:10.1186/s12889-024-18630-7)
Supplement: Supplementary file 1 — Supplementary Material 1. [file 12889_2024_18630_MOESM1_ESM.docx]

**Supplementary Table 1: Various indicators by smoking status or quitting duration**

| **Indicators** | **mg/percent/kcal (95% CI)** |
| --- | --- |
| **Vitamin C from supplement^a^ (mg)** |  |
| Current smoker | 228.6 (177.9-279.2) |
| Former smoker | 244.7 (222.3-267.0) |
| Never smoker | 224.2 (207.6-240.7) |
| **Low salt diet^b^ (%)** |  |
| Current smoker | 1.6 (1.0-2.2) |
| Former smoker | 2.4 (1.9-2.8) |
| Never smoker | 1.5 (1.3-1.7) |
| **Salt used in food preparation^c^ (%)** |  |
| Current smoker | 40.6 (38.7-42.5) |
| Former smoker | 34.5 (32.6-36.4) |
| Never smoker | 37.1 (35.9-38.3) |
| **Salt used at table^d^ (%)** |  |
| Current smoker | 36.0 (32.7-39.2) |
| Former smoker | 37.0 (34.2-38.0) |
| Never smoker | 29.2 (27.2-31.2) |
| **Obesity (%)** |  |
| Quit for 0-1 year | 38.7 (33.0-44.3) |
| Quit for <1-5 years | 42.3 (37.3-47.3) |
| Quit for <5-10 years | 37.1 (32.0-42.1) |
| Quit for <10-20 years | 43.3 (39.0-47.6) |
| Quit for <20-30 years | 42.7 (38.8-46.7) |
| Quit for <30+ years | 37.5 (33.8-41.3) |
| **>150 mins moderate to intensity work (%)** | |
| Quit for 0-1 year | 43.2 (36.2-50.1) |
| Quit for <1-5 years | 39.1 (34.0-44.2) |
| Quit for <5-10 years | 32.2 (27.1-37.2) |
| Quit for <10-20 years | 34.0 (29.7-38.3) |
| Quit for <20-30 years | 30.0 (25.9-34.0) |
| Quit for <30+ years | 27.1 (23.5-30.7) |
| **Total energy^e^ (kcal)** |  |
| Quit for 0-1 year | 2076.4 (1924.4-2228.3) |
| Quit for <1-5 years | 2110.4 (1974.5-2246.3) |
| Quit for <5-10 years | 2133.8 (1997.8-2269.8) |
| Quit for <10-20 years | 2086.4 (2010.4-2162.4) |
| Quit for <20-30 years | 2137.6 (2019.1-2256.1) |
| Quit for <30+ years | 2111.0 (1973.4-2248.7) |

* “a” represents total vitamin C intake from supplements in the past 30 days; “b” refers to adherence to a special diet - low salt/sodium diet; “c” presents the additional of any ordinary salt or seasoned salt added in cooking or preparing foods in household. “d” indicates adding any salt in meals at the table at least one day in the past two days. “e” adjusts for age, gender, race, education level, income ratio, marital status, moderate-to-intensity work, and depression status.

**Supplementary Table 2: Nutrients intake and food variety among current, former, and never smokers**

| **HEI and components** | **Current smoker** | **Former smoker** | **Never smoker** |
| --- | --- | --- | --- |
| Total Energy (kcal) | 2127.4 (2082.0-2172.8) | 2055.8 (2005.1-2106.4) | 2064.4 (2027.4-2101.3) |
| Carbohydrates (gm) | 253.3 (246.4-260.1) | 242.7 (235.3-250.2) | 253.5 (247.8-259.2) |
| Protein (gm) | 77.1 (74.8-79.3) | 80.8 (78.7-82.8) | 81.2 (79.3-83.1) |
| Total Fat (gm) | 79.1 (76.6-81.7) | 80.6 (77.9-83.4) | 79.5 (77.5-81.6) |
| Total saturated fatty acids (gm) | 25.9 (24.8-27.0) | 25.4 (24.4-26.4) | 25.4 (24.6-26.1) |
| Total mono fatty acid (gm) | 27.7 (26.8-28.6) | 28.6 (27.5-29.7) | 27.9 (27.1-28.6) |
| Total poly fatty acid (gm) | 18.0 (17.4-18.7) | 19.2 (18.4-20.1) | 18.8 (18.1-19.4) |
| Fiber (gm) | 14.5 (13.9-15.1) | 17.2 (16.5-17.9) | 17.4 (16.8-17.9) |
| Number of Foods | 13.8 (13.4-14.1) | 15.4 (15.1-15.8) | 15.1 (14.8-15.4) |

Adjusted for age, gender, race, education level, income ratio, marital status, physical activity, and depression status.
